# The Improvement in Hydrogen Storage Performance of MgH_2_ Enabled by Multilayer Ti_3_C_2_

**DOI:** 10.3390/mi12101190

**Published:** 2021-09-30

**Authors:** Zhaojie Wu, Jianhua Fang, Na Liu, Jiang Wu, Linglan Kong

**Affiliations:** 1 Department of Petroleum, Oil and Lubricants, Army Logistic Academy of PLA, Chongqing 401331, China; georgewu2019@163.com (Z.W.); wujiang179@163.com (J.W.); 2 Department of Basic Courses, Army Logistic Academy of PLA, Chongqing 401331, China; kllvj1604@163.com

**Keywords:** hydrogen storage, magnesium hydride, Ti_3_C_2_ MXene, catalyst

## Abstract

MgH_2_ has become a hot spot in the research of hydrogen storage materials, due to its high theoretical hydrogen storage capacity. However, the poor kinetics and thermodynamic properties of hydrogen absorption and desorption seriously hinder the development of this material. Ti-based materials can lead to good effects in terms of reducing the temperature of MgH_2_ in hydrogen absorption and desorption. MXene is a novel two-dimensional transition metal carbide or carbonitride similar in structure to graphene. Ti_3_C_2_ is one of the earliest and most widely used MXenes. Single-layer Ti_3_C_2_ can only exist in solution; in comparison, multilayer Ti_3_C_2_ (ML-Ti_3_C_2_) also exists as a solid powder. Thus, ML-Ti_3_C_2_ can be easily composited with MgH_2_. The MgH_2_+ML-Ti_3_C_2_ composite hydrogen storage system was successfully synthesized by ball milling. The experimental results show that the initial desorption temperature of MgH_2_-6 wt.% ML-Ti_3_C_2_ is reduced to 142 °C with a capacity of 6.56 wt.%. The E_a_ of hydrogen desorption in the MgH_2_-6 wt.% ML-Ti_3_C_2_ hydrogen storage system is approximately 99 kJ/mol, which is 35.3% lower than that of pristine MgH_2_. The enhancement of kinetics in hydrogen absorption and desorption by ML-Ti_3_C_2_ can be attributed to two synergistic effects: one is that Ti facilitates the easier dissociation or recombination of hydrogen molecules, while the other is that electron transfer generated by multivalent Ti promotes the easier conversion of hydrogen. These findings help to guide the hydrogen storage properties of metal hydrides doped with MXene.

## 1. Introduction

Energy is necessary for the survival and development of human society. In recent years, energy crises and environmental pollution have become increasingly serious with the rapid development of the global economy. The development of new energy is an important means by which to remit the contradiction between economic development and environmental protection. As an ideal secondary energy source, hydrogen energy shows many outstanding advantages, such as a high energy density of 142 MJ/kg [1], a wide range of potential sources, light weight, and environmental friendliness.

Considering that the gaseous-state hydrogen storage system shows lower safety and poor hydrogen storage capacity, metal hydrides possess attractive application prospects. Magnesium hydride (MgH_2_) shows a high theoretical hydrogen storage capacity of 7.6 wt.%, with the benefits of high capacity, abundant sources, low price, light weight, no pollution, etc. In this regard, MgH_2_ can be developed with good value as a kind of solid hydrogen storage material [2]. As an ionic compound, the adsorption and desorption process of MgH_2_ involves the formation and fracture of chemical bonds between hydrogen and metal elements, as well as crystal structure changes. Therefore, the dehydrogenation temperature of MgH_2_ is higher than 300 °C. In addition, the adsorption and desorption kinetics of MgH_2_ are poor, resulting in the slow reaction rate of absorption and desorption. The activation energy (*E*_a_) of MgH_2_ is ~143.0–160.6 kJ/mol [3,4,5,6,7]. Therefore, improving the thermodynamic and kinetic properties of hydrogen absorption/desorption reactions of Mg-based hydrogen storage materials is the key aim of current research.

In order to improve the adsorption and desorption performance of MgH_2_ hydrogen storage materials, researchers have modified MgH_2_ by alloying [8,9,10,11,12], nanoscaling [13,14,15,16,17,18,19,20], surface modification [21], and catalyst doping [22,23,24,25,26,27,28], among others. The addition of a catalyst can significantly reduce the energy barrier of hydrogen absorption and desorption reactions, thus decreasing the reaction temperature and improving the kinetic performance. Among them, Ti-based catalysts [29,30,31,32,33] can effectively improve the hydrogen absorption and desorption characteristics of MgH_2_, which has received widespread attention.

MXene is a novel two-dimensional transition metal carbide or carbonitride similar in structure to graphene, which was first synthesized by Gogotsi and Barsoum in 2011 via HF selective etching from its precursor MAX phase [34]. Because of the weak binding force between the A-MX lamellas in the MAX phase, MXene can be eroded from the A atomic layer in the MAX phase with the selection of appropriate etching agents (such as HF, LiF+HCl, NH_4_HF_2_, etc.) [35]. The general formula of an MXene is expressed as M_n+1_X_n_T_x_, in which T_x_ represents the functional groups (–OH, –F, =O, etc.) attached to the surface of the MXene, produced by chemical etching of the precursor MAX phase. At present, dozens of different components of MXenes have been successfully synthesized. As one of the earliest developed MXenes, Ti_3_C_2_ has attracted wide attention in the fields of lubricants [36,37], environmental pollution control [38], energy storage [39,40,41], and wave absorption [41,42], among others, due to its unique physical and chemical properties. In recent years, many scholars have used MXenes to improve the hydrogen absorption and desorption performance of hydrogen storage materials, especially Ti_3_C_2_ MXene. Sheng et al. [43] tried to use (Ti_0.5_V_0.5_)_3_C_2_ to reduce the initial temperature of the hydrogen desorption of MgH_2_ to 210 °C. MgH_2_+10 wt.% of (Ti_0.5_V_0.5_)_3_C_2_ can release hydrogen of 7.0 wt.% at 245 °C, and can absorb 4.8 wt.% of hydrogen at a constant temperature of 120 °C. It was shown that MgH_2_ reacted with (Ti_0.5_V_0.5_)_3_C_2_ to form Ti and V metals, which were suggested to act as active catalysts for the hydrogen sorption process. Gao et al. [44] synthesized a sandwich-like Ti_3_C_2_/TiO_2_ via partial oxidation of Ti_3_C_2_ MXene. The MgH_2_+5 wt.% of Ti_3_C_2_/TiO_2_ can release 5.0 wt.% of hydrogen at a constant temperature of 250 °C, and can absorb 4.0 wt.% of hydrogen at a constant temperature of 125 °C. The layered structures and the Ti-containing compounds with multiple valences were considered to be responsible for the improvement of MgH_2_ by Ti_3_C_2_/TiO_2_. Liu et al. [45] synthesized V_2_C and Ti_3_C_2_ MXenes by exfoliating V_2_AlC and Ti_3_AlC_2_. MgH_2_+10 wt.% of 2V_2_C/Ti_3_C_2_ initiated hydrogen desorption at around 180 °C, and 5.1 wt.% of hydrogen was desorbed within 60 min at 225 °C. Hydrogen atoms or molecules may preferentially transfer through the MgH_2_/V_2_C/Ti_3_C_2_ triple-grain boundaries during the desorption process, and through the Mg/Ti_3_C_2_ interfaces during the absorption process. V_2_C and Ti_3_C_2_ mainly act as efficient catalysts for MgH_2_ at the same time. Gao et al. [46] synthesized a few-layer Ti_3_C_2_T_x_ supporting highly dispersed nano-Ni particles through a self-assembly reduction process. MgH_2_-5 wt.% Ni_30_/FL-Ti_3_C_2_T_x_ can release approximately 5.83 wt.% hydrogen within 1800 s at 250 °C, and can absorb 5 wt.% hydrogen within 1700 s at 100 °C. This superb hydrogen storage performance was attributed to the combined effects of finely dispersed nano-Ni grown in situ on FL-Ti_3_C_2_T_x_, the large specific area of FL-Ti_3_C_2_T_x_, multivalent Ti derived from FL-Ti_3_C_2_T_x_, and the electronic interaction between Ni and FL-Ti_3_C_2_T_x_. Chen et al. [47] introduced Ti_3_C_2_ into a 4MgH_2_-LiAlH_4_ composite; the dehydrogenation onset temperature of the 4MgH_2_-LiAlH_4_-Ti_3_C_2_ composite was decreased by 64 K and 274 K with 4MgH_2_-LiAlH_4_ and with as-milled MgH_2_, respectively. The destabilization of 4MgH_2_-LiAlH_4_ can be ascribed to the Ti formed in situ from the MXene Ti_3_C_2_. Few-layer Ti_3_C_2_ can only exist in solution in the form of film, and is easy to agglomerate, which reduces the number of active sites of hydrogen absorption and desorption. In comparison, multilayer Ti_3_C_2_ (ML-Ti_3_C_2_) can exist in the form of a solid powder, which makes it easier to composite with MgH_2_. Therefore, ML-Ti_3_C_2_ may improve the hydrogen absorption and desorption performance of MgH_2_.

## 2. Experimental Details

### 2.1. Preparation of Material

Multilayer Ti_3_C_2_ MXene (ML-Ti_3_C_2_) was prepared by selective etching of Al atoms in Ti_3_AlC_2_ with an HF/HCl etching agent. The main operation methods were as follows: (1) Preparation of the etching agent: Mixing and stirring 12 mL of HCl (concentration 35–38 wt.%), 2 mL HF (concentration 49 wt.%), and 6 mL deionized water. (2) Etching: 1 g of Ti_3_AlC_2_ was slowly added to the mixed solution at 35 °C and stirred at 400 rpm for 24 h. (3) Washing: After etching, the suspension was centrifuged at 3500 rpm for 5 min to achieve the precipitation of multilayer MXene. The precipitation was washed with deionized water 5–6 times until the pH of the supernatant was ≥6, and then the precipitate was collected. (4) Drying: The collected wet powder was placed in the refrigerator for freezing, then placed in the vacuum freeze-drying oven for 24 h. The water between the layers of MXene was frozen into ice, which led to an increase in the layer spacing. In the vacuum freeze-drying oven, the frozen ice directly sublimated in vacuum to prevent the collapse of the interlayer structure. The lyophilized layers of the MXene were well spaced and accordion-like.

The as-synthesized Ti_3_C_2_ was introduced into MgH_2_ by ball milling. Experimentally, 1 g of MgH_2_ (98%, Lanabai Pharmaceutical Chemical Co. Ltd., Wuhan, China) was mixed into the milling jar with the ML-Ti_3_C_2_ in different proportions (MgH_2_+*x* wt.% ML-Ti_3_C_2_, *x* = 4, 6, 8, 10) for ball milling. Argon was used in the milling jar as the protective gas. Ball milling was carried out by all-directional planetary ball mill (PMQ0.4L, Zhuodi Instrument and Equipment Co. Ltd., Shanghai, China) at 400 rpm for 24 h; the ball-to-powder ratio was 30:1. For comparison with the former, 1 g of pristine MgH_2_ was ball milled under the same conditions. The whole experimental process was carried out under strict air isolation conditions.

### 2.2. Characterization Methods

The phase and structure analysis of samples were tested by X-ray diffractometer (XRD, DX-2700B, Hao Yuan Instrument Co., LTD, Dandong, China). The Cu Kα radiation was used for the incident ray (40 kV, 200 mA) in step scan, with a step length of 0.02 °/s and a sampling time of 1 s. Scanning electron microscopy (SEM, Regulus 8230, Hitachi Manufacturing Co. LTD, Tokyo, Japan) and transmission electron microscopy (TEM, JEM-F200, JEOL, Tokyo, Japan) were used to observe the particle size and microstructure of the samples. A microgrid copper mesh was used to hold the samples in the TEM observations. Energy-dispersive spectrometry (EDS, JED-2300T, JEOL, Tokyo, Japan and GENESIS 2000XMS, Hitachi Manufacturing Co. LTD, Tokyo, Japan) coupled with the TEM and SEM was used to analyze the micro-area composition. X-ray photoelectron spectroscopy (XPS, Thermo Scientific K-Alpha+, Thermo Fisher Scientific, Waltham, MA, USA) was utilized to analyze the chemical environments of atoms before and after the experiments. A differential scanning calorimeter (DSC, TGA/DSC2, Mettler-Toledo group, Zurich, Switzerland) was used to study the thermal behavior in hydrogen desorption. The samples were heated from room temperature to 500 °C in an argon atmosphere (20 mL/min) at rates of 5, 7, 9, and 11 °C/min.

### 2.3. De/Hydrogenation Characterization

The hydrogen absorption and desorption tests were carried out on a Sieverts-type apparatus (Institute of Metal Materials, Zhejiang University, Zhejiang, China). The apparatus was composed of a temperature-controlled tubular furnace, tubular reactors, high-/low-pressure sensors, temperature sensors, connecting pipes, and a test computer. The amount of hydrogen absorption and desorption of the sample was calculated according to the ideal gas state equation. Experimentally, the sample was weighed to ~100 mg in the glove box (H_2_O ≤ 0.01 ppm and O_2_ ≤ 0.01 ppm) each time. During the non-isothermal desorption tests, the sample was heated from room temperature to 400 °C at 2 °C/min at an initial back pressure of 10^−4^ MPa. During the isothermal absorption tests, the initial hydrogen pressure of 4 MPa was synchronously filled into the reactor, and the sample after hydrogen desorption was heated from room temperature to 300 °C at a heating rate of 2 °C/min. During the isothermal desorption tests, the sample was first heated from room temperature to the target temperature at a rate of 5 °C/min and held for 10 min, and then the valve of the connecting line was quickly opened and kept open for 1 h. During the isothermal absorption tests, the sample was first heated from room temperature to the target temperature at a rate of 5 °C/min and held for 10 min, and then the sample holder was quickly filled with hydrogen at a pressure of 4 MPa and maintained for 1 h. The quantitative information of the experimental details is shown in Table S1.

## 3. Results and Discussion

### 3.1. Characterization of ML-Ti_3_C_2_

**Figure 1:**
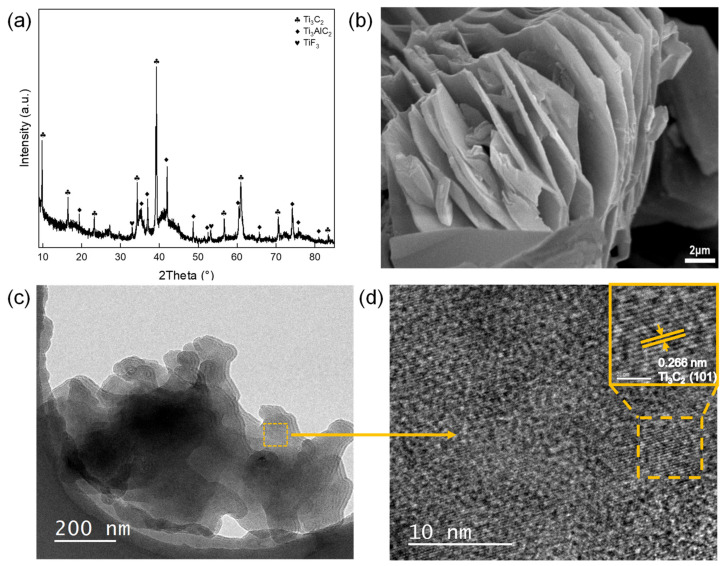
Characterizations of the as-synthesized ML-Ti_3_C_2_: (**a**) X-ray diffractometer (XRD) patterns of as-synthesized ML-Ti_3_C_2_, (**b**) Scanning electron microscopy (SEM) images, (**c**) transmission electron microscopy (TEM) image, and (**d**) High resolution TEM image.

ML-Ti_3_C_2_ was successfully obtained by selectively etching the Al layers from Ti_3_AlC_2_. Figure 1a shows the XRD patterns of as-synthesized Ti_3_C_2_ MXene. Through HF etching, the Al lamellas in the precursor MAX were effectively removed. However, the Ti_3_AlC_2_ diffraction peak still existed, indicating that the Al lamellas were not completely removed. Thus, the as-synthesized MXene was a mixture of Ti_3_C_2_ and Ti_3_AlC_2_. In addition, Ti and the F element in HF formed the TiF_3_ compound. As is shown in Figure 1b, the sample showed an accordion-like multilayer structure of multilayer MXene, with particle sizes ranging from 10 to 15 microns. EDS mapping was performed to observe the element contents, as outlined in Figure S1. EDS mapping shows that the Ti and C elements were distributed uniformly, but residual Al remained in this material, which is consistent with XRD patterns. The higher content of C may be caused by the sample table of the SEM. The presence of O may arise from the oxidation of Ti_3_C_2_ or the oxygen-containing functional groups formed after HF etching [48]. Figure 1c displays the TEM image of Ti_3_C_2_, in which the lamellar structure of ML-Ti_3_C_2_ can be seen. To further observe the microstructure of ML-Ti_3_C_2_, high-resolution TEM (HRTEM) was performed, as shown in Figure 1d. The calculated interplanar spacing of 0.265 nm is consistent with the (101) crystal planes of Ti_3_C_2_. Therefore, the XRD, SEM, and HRTEM results all confirm the successful synthesis of the ML-Ti_3_C_2_ MXene.

### 3.2. De/Hydrogenation Performance of MgH_2_+ML-Ti_3_C_2_

**Figure 2:**
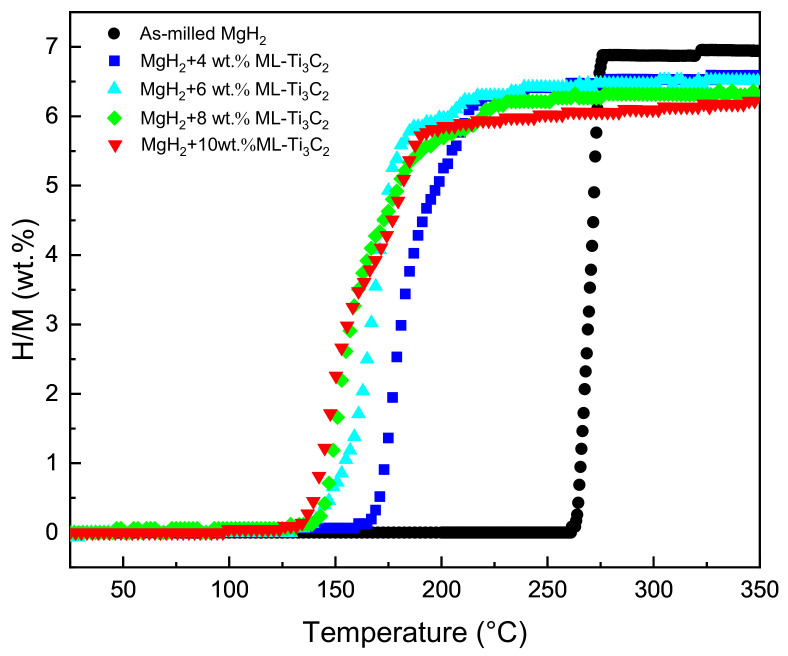
Hydrogen desorption performances of MgH_2_+*x* wt.% ML-Ti_3_C_2_ (*x* = 0, 4, 6, 8, 10).

The as-synthesized ML-Ti_3_C_2_ was introduced into MgH_2_ through ball milling to promote the de/hydrogenation performance. The prepared material systems were subjected to non-isothermal hydrogen desorption tests in order to select the best amount of ML-Ti_3_C_2_ to add. In contrast, as-milled MgH_2_ was also tested. Figure 2 displays the non-isothermal hydrogen desorption curves of MgH_2_+*x* wt.% ML-Ti_3_C_2_, (*x* = 0, 4, 6, 8, 10). The as-milled MgH_2_ begins to release hydrogen at around 267 °C, with a hydrogen desorption capacity of 7.0 wt.%. After the addition of ML-Ti_3_C_2_, the initial and the peak hydrogen desorption temperatures of the material systems were significantly reduced. With the increase of the amount of ML-Ti_3_C_2_, the initial dehydrogenation temperature decreases from 182 °C to 137 °C; however, the hydrogen desorption capacity is gradually weakened. The hydrogen desorption temperature and capacity of the material systems are shown in Table S2. When *x* = 6, the hydrogen desorption temperature reaches 142 °C, which is ~125 °C lower than that of as-milled MgH_2_. Continuing to add ML-Ti_3_C_2_, the initial dehydrogenation temperature decreases inconspicuously. MgH_2_-6 wt.% ML-Ti_3_C_2_ can also fully release its hydrogen when the temperature increases to 227 °C, showing the best overall hydrogen desorption capacity. Therefore, in subsequent experiments, MgH_2_-6 wt.% ML-Ti_3_C_2_ was taken as the object to discuss its hydrogen absorption and desorption performance.

**Figure 3:**
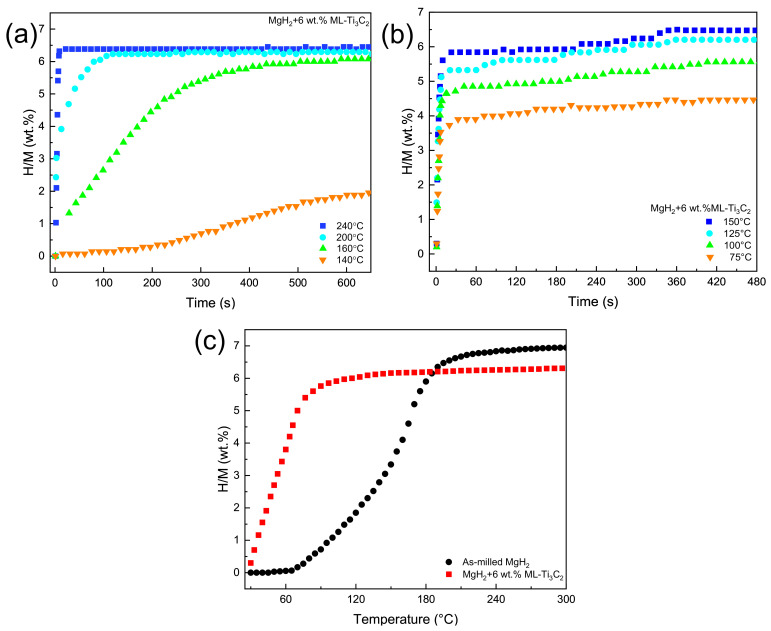
De/hydrogenation performance curves of MgH_2_-6 wt.% ML-Ti_3_C_2_ and as-milled MgH_2_. (**a**) Isothermal dehydrogenation curves at different temperatures. (**b**) Isothermal rehydrogenation curves at different temperatures. (**c**) Non-isothermal absorption curves of two samples.

To further illustrate the optimization of the dehydrogenation kinetics of MgH_2_ by Ti_3_C_2_, isothermal dehydrogenation of MgH_2_-6 wt.% ML-Ti_3_C_2_ was performed at different temperatures. Figure 3a displays dehydrogenation kinetics curves of MgH_2_-6 wt.% ML-Ti_3_C_2_ at 240 °C, 200 °C, 160 °C, and 140 °C. It is apparent that this sample possesses excellent dehydrogenation kinetics performance at 240 °C, with a hydrogen desorption capacity of 6.45 wt.% in only 10 min. In contrast, as-milled MgH_2_ does not release hydrogen at same temperature. With the decrease in the test temperature, the initial hydrogen desorption rate of the sample decreases gradually. At 140 °C, there is still 1.95 wt.% of hydrogen that can be released in 10 min, but only 3.63 wt.% can be released in 60 min after extending the test time (Figure S2).

In order to study the effect of ML-Ti_3_C_2_ on the hydrogen absorption performance of MgH_2_, the non-isothermal hydrogen absorption of the MgH_2_-6 wt.% ML-Ti_3_C_2_ system was first tested (with as-milled MgH_2_ as a control group). Figure 3c displays the absorption curves of two samples. It should be noted that MgH_2_-6 wt.% ML-Ti_3_C_2_ system shows excellent hydrogen absorption performance, immediately beginning to absorb hydrogen at room temperature (6.3 wt.%). However, the as-milled MgH_2_ after dehydrogenation does not react until 70 °C. The hydrogen absorption temperature of the sample with ML-Ti_3_C_2_ is reduced by 35 °C. The initial hydrogenation temperature and hydrogen absorption capacity of two samples are outlined in Table S3. Figure 3b displays the isothermal hydrogen absorption curves of the MgH_2_-6 wt.% ML-Ti_3_C_2_ system at temperatures of 150 °C, 125 °C, 100 °C, and 75 °C. All of the curves show excellent hydrogen absorption kinetics, with 150 °C being the best and 75 °C the worst, which reaches more than 80% of the saturated hydrogen absorption capacity of the corresponding temperature within 60 s. In addition, at lower temperatures (75 °C and 100 °C), the hydrogen absorption capacity of the MgH_2_-6 wt.% ML-Ti_3_C_2_ system reaches 4.20 wt.% and 4.86 wt.%, respectively. The initial hydrogenation temperature and de/hydrogenation capacity of MgH_2_-6 wt.% ML-Ti_3_C_2_ are outlined in Table 1. Overall, adding ML-Ti_3_C_2_ to MgH_2_ effectively improves the kinetics of hydrogen adsorption and desorption.

### 3.3. Kinetics and Thermodynamics of Hydrogen Desorption

**Figure 4:**
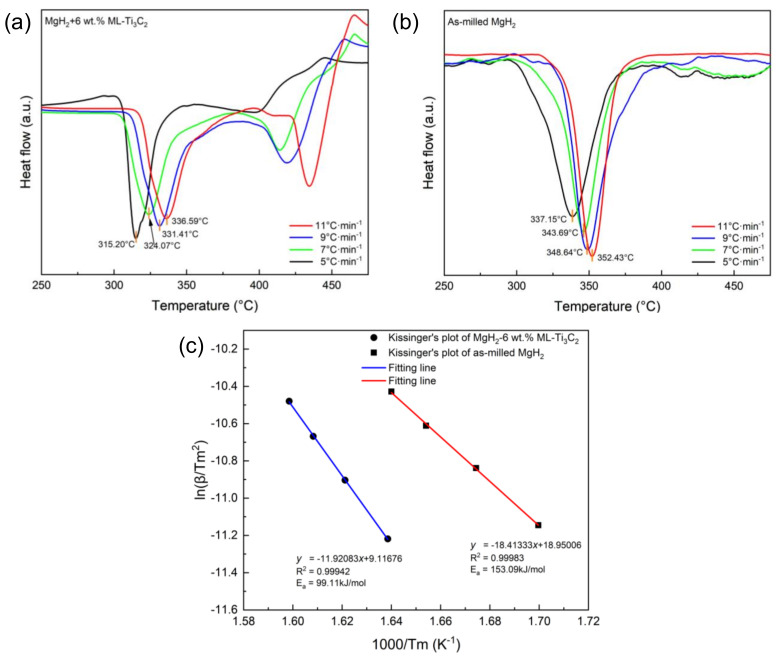
Differential scanning calorimeter (DSC) curves of (**a**) MgH_2_-6 wt.% ML-Ti_3_C_2_ and (**b**) as-milled MgH_2_ at various heating rates. (**c**) Kissinger’s plots and corresponding fitting lines for MgH_2_-6 wt.% ML-Ti_3_C_2_ and as-milled MgH_2_.

DSC analyses were further used to investigate the impact of ML-Ti_3_C_2_ MXene on the dehydrogenation kinetics thermodynamics of MgH_2_. Figure 4a,b show the DSC profiles of MgH_2_-6 wt.% ML-Ti_3_C_2_ and as-milled MgH_2_, respectively, at different heating rates (5, 7, 9, and 11 °C/min). With an increase in the heating rate, the hydrogen decomposition peaks shift to higher temperatures. The decomposition of MgH_2_-6 wt.% ML-Ti_3_C_2_ presents two thermal events, which may be caused by uneven grain size [49,50]. Furthermore, the Kissinger equation [51] was utilized to estimate the decomposition energy barrier (*E*_a_) of MgH_2_. The Kissinger equation is as follows:

ln (β/*T*_m_^2^) = −*E*_a_/R*T*_m_ + A (1)

where β represents the heating rate used in the DSC tests, *T*_m_ represents the peak temperature in the DSC curves, *E*_a_ represents the activation energy, R represents the universal gas constant, and A is also a constant. Figure 4c displays Kissinger’s plots and the corresponding fitting lines of MgH_2_-6 wt.% ML-Ti_3_C_2_ and as-milled MgH_2_. The fitting equations obtained by the Kissinger equation are as follows:

*y* = (−11.92038 ± 0.20321)*x* + (9.11676 ± 0.33879) (2)

*y* = (−18.41333 ± 0.1712)*x* + (18.95006 ± 0.27678) (3)

The *E*_a_ of dehydrogenation, obtained by the Kissinger equation, is 99.11 ± 1.69kJ/mol and 153.09 ± 1.42 kJ/mol for the composite system and the as-milled MgH_2_ respectively. It should be noted that the composite system reduces the activation energy by 35.3%. The addition of ML-Ti_3_C_2_ makes the hydrogen desorption in the two steps of the system shift to lower temperatures, but the temperature of the first step decreases more greatly. The hydrogen desorption peak in the second step widens noticeably, indicating that Ti_3_C_2_ improves the first desorption of MgH_2_ more greatly [52]. In addition, after the integral of the DSC curve, the enthalpy change of the MgH_2_-6 wt.% ML-Ti_3_C_2_ and the as-milled MgH_2_ is approximately 75.46 kJ/mol H_2_ and 78.91 kJ/mol H_2_, respectively. Therefore, the addition of Ti_3_C_2_ does not obviously improve the thermodynamic properties. This could be associated with the fact that the introduction of multilayer Ti_3_C_2_ leads neither to significant particle size refinement of MgH_2_ nor to the formation of any kind of solid solution with Ti [53]. The lower dehydrogenation temperature of the composite can effectively contribute to the kinetic improvement of MgH_2_ via the addition of ML-Ti_3_C_2_.

### 3.4. The Mechanisms for Improving the Hydrogen Storage Properties

**Figure 5:**
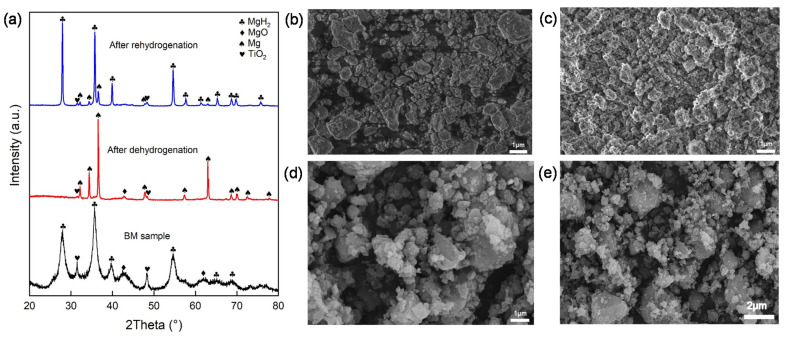
(**a**) XRD patterns of as-milled, dehydrogenative, and rehydrogenative MgH_2_-6 wt.% ML-Ti_3_C_2_. (**b**) SEM image of MgH_2_-6 wt.% ML-Ti_3_C_2_ after ball milling. (**c**) SEM image of MgH_2_-6 wt.% ML-Ti_3_C_2_ after dehydrogenation. (**d**,**e**) SEM images of MgH_2_-6 wt.% ML-Ti_3_C_2_ after rehydrogenation.

To study the mechanisms for improving the hydrogen storage properties of MgH_2_ via the addition of ML-Ti_3_C_2_, the microstructures, morphologies, and valence states of the elements were further analyzed via XRD, SEM, EDS, TEM, and XPS. Figure 5a displays the XRD patterns of as-milled, dehydrogenative, and rehydrogenative MgH_2_-6 wt.% ML-Ti_3_C_2_. After ball milling, the MgH_2_ phase shows obvious peak broadening and decreased diffraction intensity due to its small particle size and poor crystallinity [54]. In addition, a small amount of MgO and TiO_2_ is produced, which may be due to the air entering the ball mill tank during ball milling. After dehydrogenation, no MgH_2_ phase can be observed, indicating that all of that phase has been converted to Mg, but a small amount of MgO impurities still exist. After rehydrogenation, most of the Mg is converted into MgH_2_, which indicates the good reversibility of the sample in the process of hydrogen absorption and desorption. It is worth noting that no C-related peak value can be detected in the XRD patterns of the three samples, which indicates that the decomposition of ML-Ti_3_C_2_ or the lack of strong crystallinity may occur during the balling process, leading to undetectable results.

Figure 5b−e display the SEM images of MgH_2_-6 wt.% ML-Ti_3_C_2_ after (5b) ball milling (5c) dehydrogenation and (5d,e) rehydrogenation. Figure S3a,b display elemental mappings after dehydrogenation and rehydrogenation. The particle sizes of ball-milled and hydrogenated samples range from 0.1 to 2 μm, and the distribution is relatively loose. As MXene is broken and reduced, Ti and C are uniformly dispersed in the MgH_2_ matrix, increasing the number of reactive sites. After rehydrogenation, the particles expand and come into close contact. This close particle contact is not conducive to the hydrogen absorption kinetics of pure MgH_2_ [45]. However, with the addition of ML-Ti_3_C_2_, hydrogen can easily be spatially transferred through the interface between MgH_2_ and ML-Ti_3_C_2_. In general, the practical catalytic efficiency for solid-phase reactions depends not only on the intrinsic properties of the catalyst, but also on the uniform distribution of the catalytic phases, and their physicochemical interactions with the reacting phases [55]. What can be observed through the electron image and corresponding elemental mappings of MgH_2_-6 wt.% ML-Ti_3_C_2_ is that the ball milling of MgH_2_ and Ti_3_C_2_ mixtures results in particle refinement and uniform dispersion of the catalytic phase, which means the Mg, Ti, and C are distributed uniformly in the sample. This uniform distribution provides enough active catalytic sites to significantly improve the hydrogen absorption kinetics of MgH_2_.

**Figure 6:**
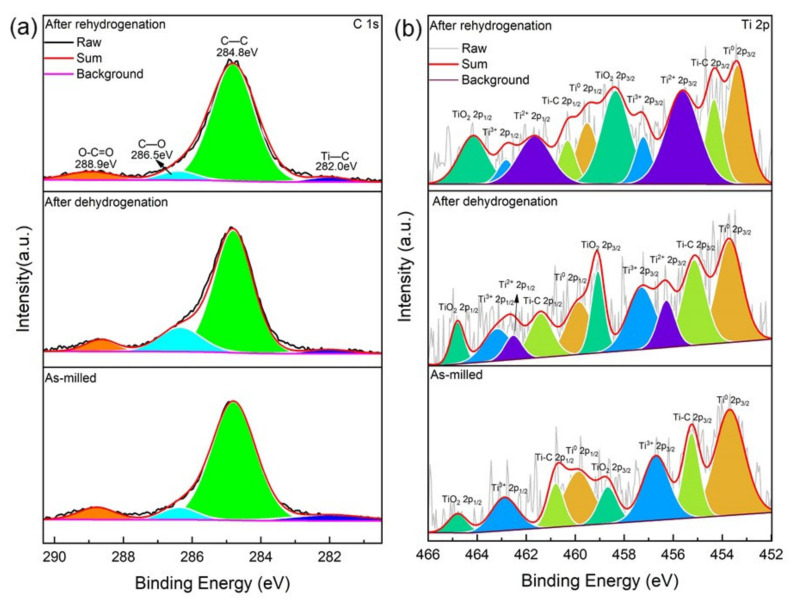
X-ray photoelectron spectroscopy (XPS) spectra of C 1s (**a**) and Ti 2p (**b**) of the as-milled, dehydrogenative, and rehydrogenative MgH_2_-6 wt.% ML-Ti_3_C_2_.

Figure 6a,b display the XPS spectra of C 1s and Ti 2p of the as-milled, dehydrogenative, and rehydrogenative MgH_2_-6 wt.% ML-Ti_3_C_2_. The C 1s XPS spectrum can be divided into four peaks: 282.0 eV, 284.8 eV, 286.5 eV, and 288.9 eV, which can be fitted to Ti−C [43], C−C [56], C−O [57], and O=C−O [57], respectively. It can be observed that Ti−C exists, hardly changes, and cannot be completely broken through ball milling, dehydrogenation, or rehydrogenation. The Ti 2p XPS spectrum after ball milling is parallel to four sets of 2p_1/2_−2p_3/2_ spin−orbit doublets at 453.7/459.8 eV, 455.1/460.9 eV, 457.0/462.2 eV, and 458.9/464.6 eV, which can be fitted to Ti^0^ [52], Ti−C [43], Ti^3+^ [58], and TiO_2_ [59], respectively. The appearance of Ti^0^ and Ti^3+^ indicates a possible chemical reduction reaction between ML-Ti_3_C_2_ and MgH_2_, which reduces Ti_3_C_2_ to Ti^0^ and Ti^3+^ during ball milling. After dehydrogenation, Ti^2+^ (456.4/461.5 eV) [57] appears, except for Ti^0^, Ti−C, Ti^3+^, and TiO_2_, indicating that the Ti^3+^ is further reduced to form Ti^2+^. After rehydrogenation, the content of Ti^2+^ increases, indicating that the reduction reaction of Ti is accompanied by the hydrogen absorption and desorption reaction.

**Figure 7:**
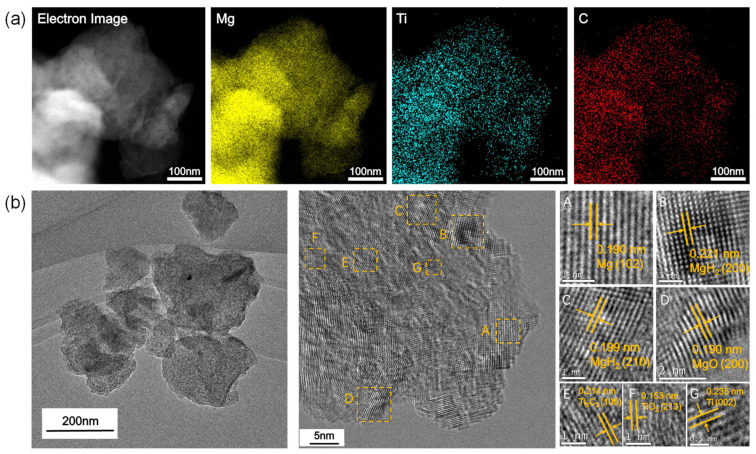
(**a**) Electron image and corresponding elemental mappings of MgH_2_-6 wt.% ML-Ti_3_C_2_ after ball milling, and (**b**) TEM and HRTEM images of the as-milled MgH_2_-6 wt.% ML-Ti_3_C_2_ (A–G: Subfigures of the middle image of Figure 2. (**b**) and corresponding interplanar spacings).

To further prove the points detailed above, Figure 7a,b show the EDS mappings of MgH_2_-6 wt.% ML-Ti_3_C_2_ after ball milling, and TEM and HRTEM images of the as-milled MgH_2_-6 wt.% ML-Ti_3_C_2_. Layered structures cannot be found via the TEM image. Combined with the EDS mappings, the microstructures of ML-Ti_3_C_2_ collapse, and are well dispersed on the MgH_2_ particles. The HRTEM analysis in Figure 7b clearly shows the different kinds of interplanar spacings (0.190, 0.221, 0.199, 0.210, 0.214, 0.153, and 0.235 nm), corresponding to the crystal planes of Mg(102), MgH_2_(200), MgH_2_(210), MgO(200),Ti(002), Ti_3_C_2_(105), TiO_2_(213), and Ti(002), respectively. Taking the TEM images, XRD patterns, and XPS spectra into account, it appears that a series of redox reactions occurred during the ball milling. A part of Ti−C fractured, Ti^3+^ and Ti^2+^ were reduced to form metallic Ti, H^−^ was oxidized into H_2_, and Mg^2+^ was reduced to form metallic Mg. In addition, on account of a small amount of O_2_ having seeped into the ball mill tank, Mg and Ti combined with O_2_ to form MgO and TiO_2_, respectively. Some reaction equations during the ball milling are as follows:

Ti^3+^ + 3e^−^ = Ti (4)

Ti^2+^ + 2e^−^ = Ti (5)

2H^−^ − 2e^−^ = H_2_ ↑ (6)

Mg^2+^ + 2e^−^ = Mg (7)

Based on the characterization analysis results above, the mechanisms by which ML-Ti_3_C_2_ improves the kinetic performance of hydrogen absorption and desorption by MgH_2_ can be preliminarily described. In the ball milling process, the zero-valent titanium formed in situ is uniformly dispersed on the surface of MgH_2_, which increases the active site of hydrogen absorption and desorption. In addition, Ti can make the hydrogen molecules on its surface easier to dissociate and recombine [60]. At the interface of MgH_2_-6 wt.% ML-Ti_3_C_2_, the displacement of Ti and Mg formed in situ in the MgH_2_ results in the deformation of MgH_2_ structure, which can destroy the Mg–H bond and generate vacancies [61]. The electron transfer caused by the change in Ti valence in the process of dehydrogenation and rehydrogenation can promote the recombination of hydrogen atoms into hydrogen molecules [33], as well as the conversion between Mg^2+^ and Mg, or between H^−^ and H_2_ [62], thus promoting the hydrogen absorption/desorption kinetics of MgH_2_. In conclusion, the enhancement of hydrogen absorption and desorption kinetics by ML-Ti_3_C_2_ can be attributed to two synergistic effects: one is that Ti facilitates the easier dissociation or recombination of hydrogen molecules, while the other is that the electron transfer generated by multivalent Ti promotes the easier conversion of hydrogen.

## 4. Conclusions

Multilayer Ti_3_C_2_ MXene was prepared by etching the precursor Ti_3_AlC_2_, and was then introduced into MgH_2_ by ball milling. The best performance of MgH_2_-x wt.% ML-Ti_3_C_2_ composite hydrogen storage materials prepared with different addition ratios reached an initial desorption temperature of 142 °C with a desorption amount of 6.56 wt.%, which is 125 °C lower than the initial desorption temperature of pristine MgH_2_. Outstanding hydrogen absorption and desorption performance indicates that the two-dimensional structure similar to that of graphene generates a large number of active sites and a high specific surface area, effectively facilitating the transport and diffusion of hydrogen in the system. The activation energy decreases from approximately 153 kJ/mol of pristine MgH_2_ to approximately 99 kJ/mol of MgH_2_-6 wt.% ML-Ti_3_C_2_—a decrease of 35.3%. DSC shows that the addition of ML-Ti_3_C_2_ does not significantly improve the thermodynamic properties, but greatly improves the kinetic properties of desorption. After dehydrogenation, hydrogen absorption easily begins at room temperature, which is 40 °C lower than that of pristine MgH_2_, while the amount of hydrogen absorption reaches 6.3 wt.%, showing good reversibility. In the ball milling process, the metallic Ti formed in situ is uniformly dispersed on the surface of MgH_2_, which increases the number of active sites of hydrogen absorption and desorption, and simultaneously promotes the dissociation of hydrogen molecules. The conversion between Mg^2+^/Mg and H^−^/H is promoted by electron transfer due to the change in Ti valence during dehydrogenation and rehydrogenation. The enhancement of hydrogen absorption and desorption kinetics by Ti_3_C_2_ can be attributed to the joint result of Ti facilitating the easier dissociation or recombination of hydrogen molecules, along with the electron transfer generated by multivalent Ti facilitating the easier conversion of hydrogen.

## Figures and Tables

**Table 1:** The initial hydrogenation temperature and de/hydrogenation capacity of MgH_2_-6 wt.% ML-Ti_3_C_2_.

Hydrogenation Temperature (°C)	Hydrogenation Capacity (wt.%)	Dehydrogenation Temperature (°C)	Dehydrogenation Capacity (wt.%)
150	6.47	240	6.45
125	6.20	200	6.29
100	4.86	160	6.08
75	4.20	140	1.95 (3.63 wt.% in 1 h)

## Supplementary Materials

The following are available online at https://www.mdpi.com/article/10.3390/mi12101190/s1, Figure S1: EDS electron image and corresponding elemental mappings of Ti_3_C_2_, Figure S2: Isothermal dehydrogenation curve at 140 °C in 60 min of MgH_2_-6 wt.% ML-Ti_3_C_2_, Figure S3: Electron image and corresponding elemental mappings of MgH_2_-6 wt.% ML-Ti_3_C_2_, Table S1: The quantitative information of the experimental details, Table S2: Initial dehydrogenation temperature and hydrogen desorption capacity of different additive amount of ML-Ti_3_C_2_, Table S3: The initial hydrogenation temperature and hydrogen absorption capacity of MgH_2_-6 wt.% ML-Ti_3_C_2_ and as-milled MgH_2_.
