# Supplementary material for: The Improvement in Hydrogen Storage Performance of MgH2 Enabled by Multilayer Ti3C2
Source: Micromachines (Basel). 2021 Sep 30;12(10):1190. doi: 10.3390/mi12101190 (PMC8541418; doi:10.3390/mi12101190)
Supplement: Supplementary file 1 [file micromachines-12-01190-s001.zip › micromachines-1357184-supplementary.pdf]

Supporting Information

The Improvement in Hydrogen Storage Performance of MgH_2 Enabled by Multilayer Ti_3C_2

Zhaojie Wu[1], Jianhua Fang [1,*], Na Liu [1,*], Jiang Wu [1] and LingLan Kong [2]

[1] Department of Petroleum, Oil and Lubricants, Army Logistic Academy of PLA, Chongqing 401331, China; georgewu2019@163.com (Z.W.); wujiang179@163.com (J.W.)

[2] Department of Basic Courses, Army Logistic Academy of PLA, Chongqing 401331, China; kllvj1604@163.com

* Correspondence: fangjianhua71225@sina.com (J.F.); liuna8911@163.com (N.L.)

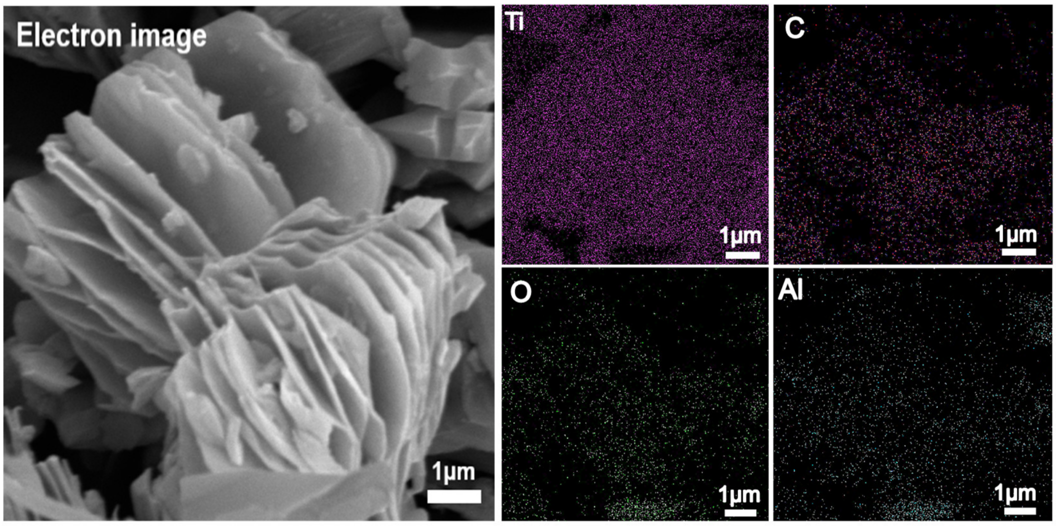

Figure S1. EDS electron image and corresponding elemental mappings of Ti_3C_2

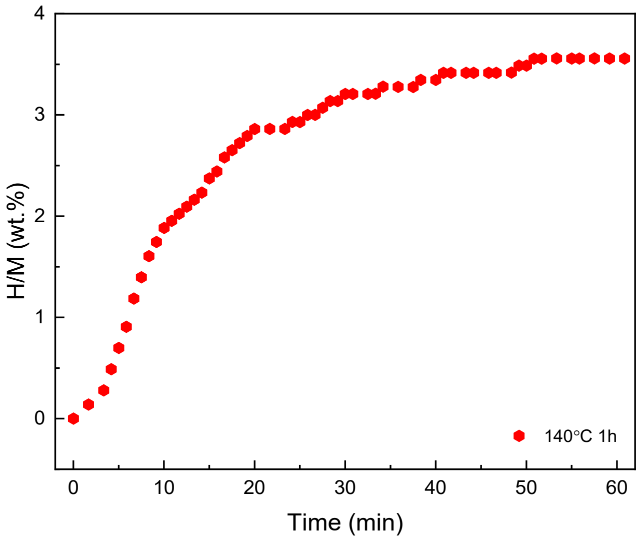

Figure S2. Isothermal dehydrogenation curve at 140 °C in 60 mins of MgH_2-6 wt.% ML-Ti_3C_2.

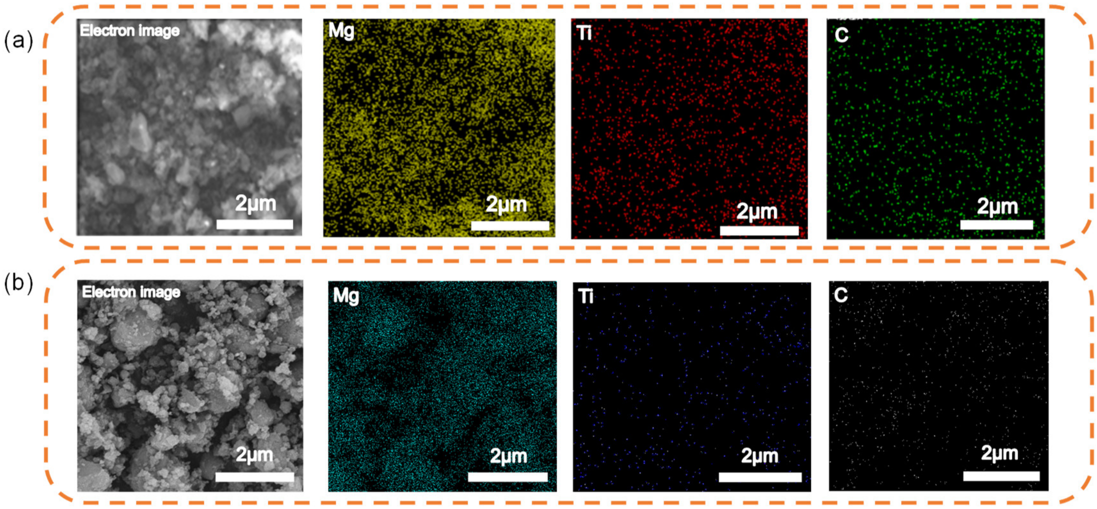

Figure S3. Electron image and corresponding elemental mappings of MgH_2-6 wt.% ML-Ti_3C_2 after (**a**) dehydrogenation and (**b**) rehydrogenation.

Table S1. The quantitative information of the experimental details.

**Experimental types**	**Sample types**	**The quality of samples (g)**	**Heating temperat-ures (°C)**	**Initial pressures (MPa)**	**Final pressures (MPa)**
Non-isothermal dehydrogenation tests	As-milled MgH_2	0.0984	RT-350	0.0001	0.0842
	MgH_2-4 wt.% ML-Ti_3C_2	0.0972	RT-350	0.0001	0.0711
	MgH_2-6 wt.% ML-Ti_3C_2	0.1020	RT-350	0.0001	0.0652
	MgH_2-8 wt.% ML-Ti_3C_2	0.0952	RT-350	0.0001	0.0621
	MgH_2-10 wt.% ML-Ti_3C_2	0.0995	RT-350	0.0001	0.0592
Non-isothermal rehydrogenation tests	As-milled MgH_2	0.0977	RT-300	4.001	3.214
	MgH_2-6 wt.% ML-Ti_3C_2	0.1032	RT-300	4.000	3.271
Isothermal dehydrogenation tests	MgH_2-6 wt.% ML-Ti_3C_2	0.1003	240	0.0002	0.0633
	MgH_2-6 wt.% ML-Ti_3C_2	0.0922	200	0.0001	0.0601
	MgH_2-6 wt.% ML-Ti_3C_2	0.0961	160	0.0000	0.0592
	MgH_2-6 wt.% ML-Ti_3C_2	0.0947	140	0.0001	0.0557
Isothermal rehydrogenation tests	MgH_2-6 wt.% ML-Ti_3C_2	0.0963	150	3.998	3.132
	MgH_2-6 wt.% ML-Ti_3C_2	0.0987	125	4.005	3.194
	MgH_2-6 wt.% ML-Ti_3C_2	0.1011	100	4.002	3.221
	MgH_2-6 wt.% ML-Ti_3C_2	0.1020	75	3.996	3.277

Table S2. Initial dehydrogenation temperature and hydrogen desorption capacity of different additive amount of ML-Ti_3C_2.

The additive amount of ML-Ti_3C_2 (wt.%)	Initial dehydrogenation temperature (°C)	Hydrogen desorption capacity (wt.%)
0	267	7.00
4	182	6.71
6	142	6.56
8	140	6.34
10	137	6.21

Table S3. The initial hydrogenation temperature and hydrogen absorption capacity of MgH_2-6 wt.% ML-Ti_3C_2 and as-milled MgH_2.

Samples	Initial hydrogenation temperature (°C)	Hydrogen absorption capacity (wt.%)
MgH_2-6 wt.% ML-Ti_3C_2	30	6.30
As-milled MgH_2	70	6.95
